# The acceptability of screening for Carbapenemase Producing *Enterobacteriaceae* (CPE): cross-sectional survey of nursing staff and the general publics’ perceptions

**DOI:** 10.1186/s13756-018-0434-x

**Published:** 2018-11-23

**Authors:** Kay Currie, Caroline King, Kareena McAloney-Kocaman, Nicola J. Roberts, Jennifer MacDonald, Adele Dickson, Shona Cairns, Nitish Khanna, Paul Flowers, Jacqui Reilly, Lesley Price

**Affiliations:** 1 School of Health & Life Sciences, Glasgow Caledonian University, Cowcaddens Road, Glasgow, Scotland G4 0BA UK; 2 NHS Health Protection Scotland, Glasgow, UK; 3 NHS Greater Glasgow & Clyde, Glasgow, UK

**Keywords:** CPE screening, Acceptability, Public perceptions, Staff perceptions, Theoretical domains framework

## Abstract

**Background:**

Carbapenemase Producing *Enterobacteriaceae* (CPE) has spread rapidly and presents a growing challenge in antimicrobial resistance (AMR) management internationally. Screening for CPE may involve a rectal swab, there are limited treatment options for affected patients, and colonised patients are cared for in isolation to protect others. These measures are sound infection prevention precautions; however, the acceptability of CPE screening and its consequences are currently unknown.

The aim of this study was **‘**To determine factors influencing acceptability of CPE screening from the perspectives of nursing staff and the general public.’

**Methods:**

National cross-sectional surveys of nursing staff (*n* = 450) and the general public (*n* = 261). The Theoretical Domains Framework (TDF) guided data collection and analysis. Regression modelling was used to identify factors that predicted acceptability of CPE screening.

**Results:**

For nursing staff, the following predictor variables were significant: intention to conduct CPE screening (OR 14.19, CI 5.14–39.22); belief in the severity of the consequences of CPE (OR 7.13, CI 3.26–15.60); knowledge of hospital policy for screening (OR 3.04, CI 1.45–6.34); preference to ask patients to take their own rectal swab (OR 2.89, CI 1.39–6.0); awareness that CPE is an organism of growing concern (OR 2.44, CI 1.22–4.88). The following predictor variables were significant for the general public: lack of knowledge of AMR (β − .11, *p* = .01); social influences (β .14**,** *p* = .032); social norms (β .21*p* = .00); acceptability of being isolated if colonised (β .22, *p* = .000), beliefs about the acceptability of rectal swabbing (β .15, p = .00), beliefs about the impact of careful explanation about CPE screening from a health professional (β .32, p = .00).

Integrating results from staff and public perspectives points to the importance of knowledge of AMR, environmental resources, and social influences in shaping acceptability.

**Conclusions:**

This is the first study to systematically examine the acceptability of CPE screening across nursing staff and the public. The use of TDF enabled identification of the mechanisms of action, or theoretical constructs, likely to be important in understanding and changing CPE related behaviour amongst professionals and public alike.

**Electronic supplementary material:**

The online version of this article (10.1186/s13756-018-0434-x) contains supplementary material, which is available to authorized users.

## Background

The World Health Organisation [1, 2] has characterised antimicrobial resistance (AMR) as a global threat to public health, with Carbapenemase Producing *Enterobacteriaceae* (CPE) identified as a growing challenge. Increases in CPE have been reported internationally, with endemic situations in the USA, India, and Greece and predictions of forthcoming worldwide epidemics [3]. In the USA, some forms of CPE infections have increased from 2 to 10% in the last decade [4]. The EUScape survey [5] highlighted the significant spread of CPE across Europe, with an endemic situation now common in the southern Mediterranean region. Within the UK, there have been isolated outbreaks reported [6]; however, significant concern exists regarding spread across geographical boundaries into areas of currently low prevalence [5]. A report published by the Centre for Disease Control pooled the results of international studies to evaluate deaths attributable to carbapenem resistant *enterobacteriaceae* infections, as opposed to carbapenem susceptible infections; the authors concluded that the number of deaths was significantly higher in resistant infections [7]. Thus, CPE is a significant and growing problem. To reduce the risk of transmission of CPE, international guidelines recommend active surveillance by rectal screening of any patient deemed ‘at risk’ of CPE carriage [8–11].

Microbiological testing for CPE normally involves obtaining a rectal swab, which may be considered more invasive and more embarrassing than testing for other organisms associated with healthcare associated infection (HAI), for example, nasal swabs for Meticillin-resistant *Staphylococcus aureus* (MRSA). Anecdotal evidence suggested that nursing staff were uncomfortable asking patients for a rectal swab to screen for CPE. In addition, there are currently no decolonisation protocols for clearing CPE and relatively few treatment options for patients should they develop a CPE infection; once admitted to hospital, patients colonised with CPE are managed in isolation for the protection of other patients. All of these factors may affect the acceptability of CPE screening from the perspectives of patients and staff. It is a well-established public health principle that if we are to expect routine implementation of any screening intervention, then it should be acceptable to all stake-holders, in this case, clinical staff implementing the CPE screening programme as well as patients being screened [12, 13]. Whilst Dyakova et al [14] have recently highlighted factors associated with patients who refused to provide a rectal swab as part of a prevalence study of ESBLs, at the time our study commenced (2016), we were unable to locate any published evidence on behavioural or attitudinal factors which influenced the acceptability of routine CPE screening from the perspectives of either nursing staff or the general public.

This paper reports findings from two national cross-sectional surveys of the acceptability of CPE screening; one from the perspective of Scottish nursing staff and the other from the general public. The aim of the study was to determine those factors which may predict acceptability of CPE screening.

## Methodology

As our interest was in exploring those factors which might influence staff and patient attitudes and behaviours related to the acceptability of CPE screening, we sought an appropriate theoretical framework from implementation science to guide study design, data collection, and analysis. The Theoretical Domains Framework enables exploration of psychosocial constructs across 14 distinct ‘domains’ and can explain an individual’s decisions to act or behave in specific situations [15, 16]. In our case, constructs from the TDF were used to develop survey items which were analysed to predict individual psychosocial factors that would influence the acceptability of CPE screening, from both nursing staff and general public perspectives. By identifying the antecedents, or influencing factors, that are central to implementing screening, it is possible to direct future behaviour change intervention development.

The study was reviewed and approved by the School of Health and Life Sciences Ethics Committee at Glasgow Caledonian University (HLS/NCH/15/18). Access permission to NHS Nursing staff was granted by all 15 Scottish Regional Board Executive Leads for healthcare associated infection.

### Data collection

Data was collected via two cross-sectional surveys (Additional file 1). Participants were asked to indicate their level of agreement with a series of statements on a Likert type scale from strongly disagree to strongly agree. The dependent variable for the clinical staff survey was “I believe that CPE screening is acceptable” and for the public survey, “If I were to be admitted to hospital I would find CPE screening acceptable”. Independent variables were aligned with relevant TDF constructs to enable subsequent interpretation of results in light of this theoretical framework. Survey tools were piloted with a small group of members of the public and with nursing educators; minor changes were made to questions to enhance ease of understanding.

A paper questionnaire was distributed via hospital based link co-ordinators to 588 nursing staff working in different clinical areas in acute hospitals expected to screen for CPE, across all 15 Scottish health boards. Three paper-based questionnaires were distributed to selected areas in every acute hospital: one to be completed by the nurse in charge and two by other nurses. Completed questionnaires were sealed, then collected and returned to the research team via the link co-ordinator.

For the public component, a paper-based questionnaire was distributed to a random sample of 1000 names selected from the open electoral rolls of four diverse geographical regions of Scotland. Given the recognised limitations of postal survey recruitment [17], an electronic questionnaire was also made available via SurveyMonkey© and distributed via email link to local University students and promoted via social media networks.

### Data analysis

Survey data was entered into SPSS (Version 23)© for analysis. For the clinical staff survey, each questionnaire item related to participants’ responses was collapsed into dichotomous variables; agree versus all other responses (do not agree, don’t know and neither). The general public questionnaire items collected responses using a 10 point scale, where scores of 1 indicated strongly disagreeing with a statement and a score of 10 indicated strong agreement. Any responses for questionnaire items using this scale were coding accordingly from 1 to 10. Additionally participants were asked to respond to a number of categorical response items about demographics, their awareness of antimicrobial resistance and CPE itself, and experiences of and preferences for screening. Descriptive analysis and univariate inferential testing (Chi-Square, Mann-Whitney, Kruskall-Wallis and correlation analyses) were undertaken for both nursing staff and general public samples, to explore relationships with the respective dependent variables (‘I believe that CPE screening is acceptable’ and ‘If I were to be admitted to hospital I would find CPE screening acceptable’), and for the purpose of variable reduction in regression modelling. For the nursing staff survey a multiple logistic model was estimated, and for the general public survey a multiple linear regression model. In the multiple logistic regression model the Wald statistic provides an indication of whether the variables in the model contribute significantly to the dependent variable. Odds ratios and 95% confidence intervals of the odds ratios are provided as an indication of the effect size of the association between the dependent and independent variables. For the multiple linear regression model the t-statistic is reported as an indication of the reliability of the regression co-efficient, and the regression co-efficient is reported as standardised beta.

Finally, the resultant significant predictor variables in each model were then mapped against TDF [15] constructs to interpret findings in light of the mechanisms important in influencing behaviour related to the acceptability of screening for CPE.

## Results

The survey response rate for nursing staff was 86% (505/588). A final sample of 450 viable responses were included in descriptive analysis. As there were 14 missing responses to the screening dependant variable questions, 436 participants were included in the inferential analysis and modelling; sample sizes met power calculation requirements (minimum sample size of 350 respondents) specifically for a logistic regression [18].

It was not possible to calculate a response rate for the general public survey as the number of recipients of the online questionnaire distributed via social media is unknown. Therefore emphasis was placed on achieving a sample size that met the power calculation requirements for the multiple linear regression. Ninety-two out of 1000 (9.2%) responses from the paper based general public survey were combined with 216 responses from the online survey, giving a total of 308 public survey responses; 47 were excluded due to incomplete data, which left a sample of 261 viable responses. This exceeded the power calculation requirements of 220 participants for the range of planned analyses [19].

### Sample characteristics

Among the nursing staff 30.7% were senior nurses, 60% were staff nurses and 9.3% health cares assistants. Clinical areas represented by the nursing staff were renal (8.7%), care of the elderly (9.3%), pre-admission (9.6%), orthopaedics (17.8%), general surgery (11.3%), general medical (17.1%), vascular (3.8%), and receiving (3.8%) the remaining 2.8% reported either another clinical areas or no clinical area was reported.

#### Acceptability of CPE screening

Whilst the majority (*n* = 303, 67.3%) of nursing staff agreed ‘I believe that CPE screening is acceptable’, perhaps more notably, 1/3 nursing staff did not agree that CPE screening is acceptable. Table 1 reports the frequencies of agreement with the CPE items for nursing staff.

**Table 1:** Frequency of agreement to survey items among nursing staff

Survey Questions	Agree N (%)
I am aware that CPE is an emerging multi-drug resistant bacteria of growing concern. (CPE1)	68.4
I have been informed about my hospital’s policy and processes for screening patients for CPE. (CPE2)	55.1
Screening for CPE is undertaken in the clinical area I work in (CPE3)	76.2
The consequences of CPE infection for the patients I care for is/will be so severe that screening will always be a priority. (CPE5)	46.9
Screening patient for CPE would be/is embarrassing for them if a rectal swab is/was required. (CPE6)	66.9
If a rectal swab is/was required as part of CPE screening for the patient I care for they should be asked to do this themselves, if they are able.(CPE7)	74.0
Screening patient for CPE is/would be embarrassing for me as I may need to ask to take a rectal swab (CPE8)	20.0
I intend to conduct CPE screening, on patients on admission, according to my hospital policy. (CPE9)	79.0
I believe that CPE screening is acceptable. (CPE10)	67.3

Among the general public, while there was a high prevalence of awareness of antibiotic resistance, there was low reported knowledge of CPE specifically. Table 2 reports descriptive statistics of the general publics’ understanding and awareness of CPE.

**Table 2:** Descriptive statistics of the general publics’ understanding and awareness of CPE

Question	N (%)
Have you heard about the problem of some bacteria becoming resistant to antibiotics	
Yes	214 (83.9)
No	41 (16.1)
Have you heard of CPE (*n* = 256)	
Yes	59 (23.0)
No	197 (77.0)
Have you been screened for CPE (n = 256)	
Yes	2 (0.8)
No	191 (74.6)
Don’t know	63 (24.6)
Have you ever had a rectal swab or rectal examination by a Doctor or Nurse? (*n* = 254)	
Yes	61 (24.0)
No	193 (76.0)
Have you ever provided a stool sample (*n* = 253)	
Yes	124 (49.0)
No	129 (51.0)
If I needed to be tested for CPE, I would prefer to provide? (*n* = 254)	
Rectal swab	34 (13.4)
Stool specimen	107 (42.1)
I have no preference	113 (44.5)
If a rectal swab was required to test you for CPE, would you prefer (*n* = 250)	
A nurse took the swab	47 (18.8)
You did this yourself	111 (44.4)
I have no preference	92 (36.8)

Median scores for the general public for the dependent variable ‘If I were to be admitted to hospital I would find CPE screening acceptable’ indicated high levels of agreement (Median = 9, IQR = 3). Similarly, high levels of general public agreement were demonstrated in relation to the acceptability of having a rectal swab taken (Median = 9, IQR = 3) or being cared for in isolation as a consequence of a positive screen (Median = 8, IQR = 4). For the general public, there were no significant differences between acceptability according to age or gender (see Table 3).

**Table 3:** Associations between age and gender and acceptability

Variable	Median (IQR)	Test Statistic	P
Gender			
Male	8.00 (3.00)	5388^a^	0.12
Female	9.00 (3.00)		
Age			
16–24	9.00 (3.00)	7.21^b^	0.07
25–40	8.00 (3.25)		
41–64	9.00 (2.00)		
65 and over	9.00 (2.00)		

^a^Mann-Whitney U test

^b^Kruskall Wallis test

#### Univariate analysis

Univariate analysis indicated that there was significant variation in the acceptability of CPE screening among nursing staff across independent variables reflecting TDF domains of ‘intentions to screen’; ‘environmental context and resources’; ‘knowledge’; ‘social influences’; ‘beliefs about the severity of consequences of CPE’; and, ‘emotion’ (embarrassment for patients in being asked for rectal swab). For the pubic data, univariate analysis revealed significant variations in acceptability of CPE screening across experience of health care employment, the acceptability of being cared for in isolation and the TDF domains of ‘knowledge’; ‘social influences’; and ‘beliefs about the severity of consequences of CPE’. All significant predictors were subsequently entered into regression models. (univariate results available in Additional file 2).

#### Results of regression modelling

The following section presents the results of the modelling for the nursing staff (Table 4) and general public (Table 5), aligned with the respective theoretical domain.

**Table 4:** Nursing staff results for logistic regression model

TDF Domain	Significant predictors			
	Questionnaire Item^a^	Wald	OR	95% CI
1. Intentions	I intend/ would intend to conduct CPE screening, on patients on admission, according to my hospital policy. (79% nursing staff agree)	26.17***	14.2	5.1–39.2
2. Beliefs about the severity of consequences	The consequences of CPE infection for the patients I care for is/will be so severe that screening will always be a priority. (47% nursing staff agree)	24.15***	7.1	3.26–15.6
3. Knowledge, and Environmental context and resources	I have been informed about my hospital’s policy and processes for screening patients for CPE. (55% nursing staff agree)	8.70**	3.04	1.45–6.3
4. Social influences	If a rectal swab is/was required as part of CPE screening for the patient I care for they should be asked to do this themselves, if they are able. (74% nursing staff agree)	8.12**	2.89	1.39–6.0
5. Knowledge	I am aware that CPE is an emerging multi-drug resistant bacterium of growing concern. (68% nursing staff agree)	6.36*	2.44	1.22–4.9

Adjusted odds ratios, controlling for Health Board and reported CPE screening undertaken in the clinical area

* = *p* < 0.05, ** = *p* < 0.01; *** = p < 0.001; Wald reported to 2 significant places [27]; OR and 95% CI reported in line with rule of four [28]

^a^In all cases ‘Not agreeing’ with the statement is the reference category

**Table 5:** General public results for linear regression model

TDF Domain	Significant predictors	Beta	t
	Questionnaire Item^a^		
1. Knowledge:	Are you aware of the problem of some bacteria becoming resistant to antibiotics	−.11*	−2.54
2. Social influences	Composite item of transmission and screening responsibility	.14*	2.16
3. Social influences	I would find rectal swabbing for CPE acceptable	.15**	2.99
4. Social influences	I believe that CPE screening would be acceptable to most people being admitted to hospital	.21***	4.03
5. Social influences	I would find being place in a single room acceptable	.22***	4.36
6. Knowledge: Social influences	Careful explanation about CPE screening from a health professional would make screening more acceptable to me	.32***	5.85

^a^Note: All predictors except the first (Knowledge) entered as scale variables; model includes controls for health care profession experience, screening experience, and TDF items identified as significant at univariate analysis but not significant in the model (full table available on request)

* = *p* < 0.05, ** = *p* < 0.01; *** = *p* < 0.001; standardised beta reported to 2 significant places [27]

For nursing staff, logistic regression analysis resulted in a well-fitting model (H-L χ^2^ = 9.96, df = 8, *p* = 0.27) accounting for 57% of the variance in the outcome (Nagelkereke R^2^). Five predictors emerged as significantly associated with acceptability in the model, in the theoretical domains: ‘intentions to screen’; ‘beliefs about the severity of consequences of CPE’; ‘environmental context and resources’; ‘social influences’; and ‘knowledge’ (Table 4).

For the general public, a multiple linear regression model explained over 68% of the variance in acceptability of CPE screening (R^2^ = 0.68), and was significant (F = 26.9, *p* < 0.001). Acceptability of CPE screening was significantly associated with six key predictors related to the theoretical domains of ‘knowledge’ and ‘social influences’ (Table 5). Not having heard of the problem of bacteria becoming resistant to antibiotics was associated with lower ratings of CPE screening acceptability. Higher scores on the social influence scale, as well as explanations from health care professionals, were associated with higher ratings of acceptability (scale items related to concern about transmission to others; beliefs about family norms; sense of responsibility to be screened). Higher acceptability ratings of items related to components of CPE management (rectal screening, isolation in single room) were also associated with higher ratings of acceptability of CPE.

## Discussion

To the authors’ knowledge, this is the first study to have investigated factors which influence the acceptability of CPE screening and management from the perspectives of both nursing staff and the general public, using the TDF. Given the global threat of CPE, and the continued emergence of new multi-drug resistant organisms, understanding those barriers and enablers which may influence the acceptability of screening is fundamental to improving screening uptake and thereby reducing the risk of CPE transmission in hospitals. Using TDF to guide the research means that influencing factors can be mapped to specific behaviour change interventions to guide future recommendations.

Whilst regression modelling identifies specific variables found to predict the acceptability of CPE screening, of particular interest to this discussion is the influence of the theoretical domains of ‘knowledge’, ‘environmental resources’, and ‘social influences’, which were common to both nursing staff and general public groups.

For both nursing staff and the general public, knowledge and awareness of AMR in general and CPE in particular were associated with greater acceptability of CPE screening. For nursing staff, knowledge may lead to greater appreciation of the potential severity of consequences of CPE infection and possibly shape attitudes towards the acceptability of screening procedures. However, fewer than half of the nursing staff respondents agreed that the consequences of CPE would be severe. If, as we found, greater acceptability is influenced by belief in the severity of consequences, then nursing staff must be supported to develop greater understanding of the potential severity of CPE. However, providing opportunities to develop staff knowledge and awareness can be challenging. Similarly, whilst the ‘intention to conduct screening in line with hospital policy and procedure’ was an important predictor in explaining staff perceptions of CPE screening acceptability, just over half of respondents agreed they had been made aware of hospital policy. This emphasises the pivotal link between ‘environmental resources’, as an enabler, and nursing staff ‘knowledge’; resources are required to facilitate staff training about CPE policies and thus influence staff attitudes towards the acceptability of screening. This finding echoes the work of Parker et al. [20], who also report the challenges in addressing low staff awareness of CPE and providing educational opportunities across intensive care units in three New York hospitals.

Knowledge about AMR was also one of the most important factors in understanding the public’s acceptability of CPE screening. The O’Neill Report [21] highlights the ‘urgent priority’ of a global public awareness campaign as a means of tackling AMR. A recent systematic review [22] considered 54 studies and concluded that the public have an incomplete understanding of antibiotic resistance, misconceptions about its causes, and do not believe they contribute to its development. However, there is some evidence from systematic reviews that by increasing awareness and knowledge of AMR, demand for and prescription of unnecessary antibiotics shows decline [23, 24]. In the context of the acceptability of CPE screening, our results indicate that knowledge of AMR enables a broader understanding of why CPE screening, and rectal swabbing in particular, is important. Therefore, increasing public knowledge about AMR more broadly is likely to increase personal acceptability of CPE screening, and associated consequences of being cared for in isolation, for patients. Thus, the issue of public awareness of AMR, in general, requires to be addressed.

The fact that ‘careful explanation of CPE screening from a healthcare professional’ also predicts personal acceptability indicates the ‘social influence’ that nurses may have on patients’ attitudes. This finding has parallels with the recent work of Dyakova et al [14], who reported a reduction in the number of research study participants who refused to consent to a rectal swab after the explanation of the procedure had been simplified to focus on providing a consistent message around potential benefits to patients and their peers. The influence of the nurse in this situation also underlines the importance of nurses having adequate knowledge themselves, in order to explain the reasons for screening to patients. This point reiterates the link between nursing ‘knowledge’ and their capability to exert ‘social influence’ in this situation.

Other social influences are at play from both staff and patient perspectives. Beliefs regarding asking patients to self-swab also made a notable contribution to explaining the variance in CPE screening acceptability for nursing staff. Staff appear to believe that the procedure is embarrassing for patients and consequently think it would be more acceptable if those patients that were able to, self-swabbed. This is a concern, as the effectiveness of rectal self-swabbing has not yet been shown to be reliable in the context of CPE screening. A study of the sensitivity of rectal versus perineal swabs for detecting ESBL *Enterobacteriaceae* [14] showed that rectal swabs were around two times more sensitive than perineal swabs for detecting the organism and that staff-collected swabs resulted in higher detection rates than patient-collected swabs. Unfortunately that study gave patients the option for staff or self-swab, but did not compare staff and self-swabbing in the same patient. Further research to assess the reliability of self-swabbing in this context is required. Conversely, despite the concerns expressed by nursing staff, the general public survey demonstrated high levels of ‘strong agreement’ that rectal swabbing was acceptable.

Wider ‘social influences’ also predicted the personal acceptability of CPE screening for the public. This variable captured people’s perceptions of social responsibility regarding screening (they felt it was their and other people’s social duty to be screened). The role of such social norms in determining behaviour is debated in the literature; for example, a meta-analysis of 196 studies undertaken by Manning [25] suggests that whilst there is a relationship between injunctive norms (a behaviour perceived to be approved of by others in a social group) and actual behaviour, this is not always strong. However, our findings suggest that providing the public with information about social norms, noting that most people find screening acceptable, as well as focusing attention on ideas of collective action or the public good, may well increase the personal acceptability of CPE screening; further research is required in this area.

Whilst views from across a range of ages and geographical distribution of the public were obtained we make no claims on the representativeness of the general public sample. However, the sample sizes did meet power calculation requirements for both nursing staff and the general public. A key strength of the study is the integration of psychological theory, in the form of TDF [15], which enables the development of targeted, psychologically based interventions. Work by Mitchie et al. [26], based on the TDF, has identified a wide range of potential interventions, or taxonomy of Behaviour Change Techniques (BCTs), which can be proposed in response to identified barriers or enablers within specific theoretical domains. This means that future interventions to address the barriers and enablers to the acceptability of CPE screening (knowledge, environmental resources, social influences) can be determined on the basis of well-established behaviour change principles.

## Conclusions

This report provides original evidence of barriers and enablers to the acceptability of CPE screening from the perspectives of nursing staff from 15 Scottish regional Health Boards and the Scottish general public. Developing knowledge and understanding of AMR, providing adequate environmental resources and capitalising on social norms to influence behaviour are all enablers which may enhance the acceptability of CPE screening.

Understanding the nature of AMR in general and CPE in particular creates the foundation for acceptability of CPE screening for both nursing staff and the public. With knowledge and appreciation of the consequences of CPE, at both local and global health levels, staff are better equipped to provide appropriate information to patients. Staff could then also appreciate the importance of reliable swabbing for microbiological testing and overcome potential socially based barriers regarding patient embarrassment. However, staff require the environmental context to deliver resources, in terms of time and training opportunities, to enable the development and sharing of this knowledge. For the public, greater knowledge regarding the importance of reducing the transmission of infection and recognition of the social influences of responsibility for self and others, would enable the acceptability of CPE screening and its consequences.

In considering both staff and general public perspectives, the concern expressed by nursing staff that patients would find being asked for a rectal swab embarrassing appears to be unfounded. This is an important message to share with nursing staff, as careful explanation about CPE screening from a healthcare professional makes CPE screening more acceptable to the public.

This study has identified the range of factors which present as barriers and enablers to the acceptability of CPE screening. Interventions based on psychological theory, such as the behaviour change wheel [26], should be adopted to systematically identify key intervention components based on the specific barriers and enablers within the domains of knowledge, social influence, and environmental resources.

## Additional files

Additional file 1: Survey tools. (PDF 788 kb)

Additional file 2: Univariate analysis. (DOCX 23 kb)
