# Supplementary material for: The acceptability of screening for Carbapenemase Producing Enterobacteriaceae (CPE): cross-sectional survey of nursing staff and the general publics’ perceptions
Source: Antimicrob Resist Infect Control. 2018 Nov 23;7:144. doi: 10.1186/s13756-018-0434-x (PMC6260859; doi:10.1186/s13756-018-0434-x)
Supplement: Supplementary file 1 — Survey tools. (PDF 788 kb) [file 13756_2018_434_MOESM1_ESM.pdf]

Nursing staff Questionnaire:

Screening for multi-drug resistant bacteria in NHS Hospitals

- You are being invited to complete a questionnaire. It asks about your views on what helps or hinders staff to carry out screening patients for multi-drug resistant bacteria.
- The questionnaire should only take **5-7 minutes** to complete.
- If you want to ask any questions about the study before deciding to take part or not please contact Dr Caroline King (0141 3318725 Caroline.King@gcu.ac.uk)

Background to the study

One way of managing the spread of multi-drug resistant bacteria in hospitals is to screen patients, on or prior to admission to hospital. *Methicillin-resistant Staphylococcus aureus* (MRSA) and Carbapenemase-producing Enterobacteriaceae (CPE) are both types of resistant bacteria for which national guidelines recommend screening. Screening for MRSA has been in place for the last decade whereas CPE screening has been introduced more recently. Screening is a two stage process involving a clinical risk assessment in which patients are asked a few questions. If the answer to any of these questions is yes, identified body sites are swabbed for laboratory analysis. For CPE this will normally include a rectal swab, or a stool sample where the swab cannot be obtained.

Health Protection Scotland has asked Glasgow Caledonian University to conduct a study. Participation in the study is voluntary and we have the approval of your Health Board and ethical approval from Glasgow Caledonian University to conduct this study. We do not ask for your name or contact details and it will not be possible to identify you from your answers. By completing and returning the questionnaire you are consenting to taking part.

The questionnaire

You may skip any questions that you do not want to answer.

To answer the questions please tick **one box only** for each question to show how much you agree or disagree with the statements. You may add any comments you have in the box provided at the end of the questionnaire.

Example answer

Young people today spend too much time playing electronic games.

Strongly disagree	Mostly disagree	Somewhat disagree	Neither agree or disagree	Somewhat agree	Mostly agree	Strongly agree	Don't know
		✓					

Section two: CPE screening. Please tick only one box per question.

1. I am aware that CPE is an emerging multi-drug resistant bacteria of growing concern.

Strongly agree	Mostly agree	Somewhat agree	Neither agree or disagree	Somewhat disagree	Mostly disagree	Strongly disagree	Don't know

2. I have been informed about my hospital's policy and processes for screening patients for CPE.

Strongly agree	Mostly agree	Somewhat agree	Neither agree or disagree	Somewhat disagree	Mostly disagree	Strongly disagree	Don't know

3. Screening for CPE is undertaken in the clinical area I work in.

Yes	No	Don't know

4. When admitting patients to your clinical area, how many times do you complete a CPE clinical risk assessment?

Every time	Usually - about 90% of the times	Frequently - about 70% of the times	Sometimes - about 50% of the times	Occasion-ally - about 30% of the times	Rarely - about 10% of the times	Never	Don't know

5. The consequences of CPE infection for the patients I care for is/will be so severe that screening will always be a priority.

Strongly agree	Mostly agree	Somewhat agree	Neither agree or disagree	Somewhat disagree	Mostly disagree	Strongly disagree	Don't know

6. Screening patients for CPE would be/is embarrassing for them if a rectal swab is/was required.

Strongly agree	Mostly agree	Somewhat agree	Neither agree or disagree	Somewhat disagree	Mostly disagree	Strongly disagree	Don't know

7. If a rectal swab is/was required as part of CPE screening for the patient I care for they should be asked to do this themselves, if they are able.

Strongly agree	Mostly agree	Somewhat agree	Neither agree or disagree	Somewhat disagree	Mostly disagree	Strongly disagree	Don't know

8. Screening patient for CPE is/would be embarrassing for me as I may need to ask to take a rectal swab.

Strongly agree	Mostly agree	Somewhat agree	Neither agree or disagree	Somewhat disagree	Mostly disagree	Strongly disagree	Don't know

9. I intend/ would intend to conduct CPE screening, on patients on admission, according to my hospital policy.

Strongly agree	Mostly agree	Somewhat agree	Neither agree or disagree	Somewhat disagree	Mostly disagree	Strongly disagree	Don't know

10. I believe that CPE screening is acceptable.

Strongly agree	Mostly agree	Somewhat agree	Neither agree or disagree	Somewhat disagree	Mostly disagree	Strongly disagree	Don't know

<u>Please continue to Section three</u>

Section three: about you and your clinical area.

Please tick only one box per question or if you select "other" please write your answer in the box provided.

1. Are you a?

Senior Charge Nurse	Staff nurse	Healthcare assistant

Other please specify

2. What type of clinical area do you work in?

Renal	Care of the elderly	Pre-admission	Surgical receiving	Medical receiving	Orthopaedics	General surgical	General medical

Vascular	Other please specify

3. Which Health Board do you work in?

NHS Ayrshire and Arran	NHS Borders	NHS Dumfries and Galloway	NHS Fife	NHS Forth Valley	NHS Grampian	NHS Greater Glasgow and Clyde	NHS Highland

NHS Lanarkshire	NHS Lothian	NHS Orkney	NHS Shetland	NHS Tayside	NHS Western Isles	Golden Jubilee Hospital

Please add any comments about MRSA or CPE screening you wish to make in the box below.

Thank you. Please return this questionnaire to the person who gave it you.

Working together in Scotland to deal with superbugs: your views

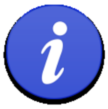

Superbugs have been in the news a lot recently. Superbugs are bacteria which can cause infections that are harder to treat with antibiotics.

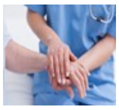

By working together with our nurses and doctors we can manage these superbugs much better.

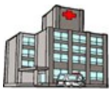

One way to do this is for our nurses and doctors to check us for these bacteria when we become patients in hospital.

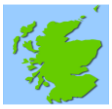

In Scotland, nurses and doctors are starting to check patients for a bug known as CPE (*Carbapenemase Producing Enterobacteriaceae)* – we want to know your views about this.

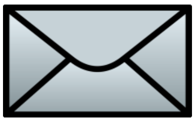

To send us your views:

- Please complete this questionnaire
- Post it to us by the **20th February** in the pre-paid envelope provided.
- You do not need to have any direct experience of superbugs to answer the questions – we are asking you because:
  - You live in Scotland
  - You may either use our hospitals at the moment or at some time in the future
- If there are any questions you would prefer not to answer, please leave them blank

First, we would like you to answer some questions about yourself. This will let us know that we have collected the views of a range of people of different ages and circumstances. We are not able to identify individual people from this information.

To answer the questions please tick one box for each question

1. Your age?

16-25	26-40	41-64	65-79	Over 80

2. Your gender?

Male	Female

3. Your employment status?

Unemployed	Self-employed	Employee	Retired	Full-time carer for family	Student

4. If employed in any capacity or retired, please state your current or past occupation?

5. Have you ever worked as a healthcare professional?

Yes	No

6. Have you been admitted to hospital in the last five years that included an overnight stay?

Yes	No

7. Are you parent for a child under 16?

Yes	No

8. Are you a carer for another dependant adult?

Yes	No

9. Your postcode? First part only to identify which part of Scotland you live in (e.g., EH12)?

The superbug we are asking about in this questionnaire is called CPE.

To answer the questions please tick one box for each question

1. Have you heard about the problem of some bacteria becoming resistant to antibiotics *(antimicrobial resistance or AMR)*?

Yes	No

2. Have you heard about CPE?

Yes	No

3. If you have heard, what was the **main** source of your information about CPE?

Social media	Newspapers or magazines	TV or radio	Internet	Posters in public places	Healthcare professional	Family, friends or colleagues

Other please specify

4. Have you ever been screened for CPE?

Yes	No	Don't know

CPE is common in some countries and so we are keen to look at ways of preventing the spread of CPE in Scotland

In this section we begin to ask questions about how strongly you agree or disagree with statements about CPE. Here is an example of how someone would respond if they disagreed to some extent with the following statement:

I believe CPE screening is beneficial to patients

	1	2	3	4	5	6	7	8	9	10	
Disagree strongly			✓								Agree strongly

To answer please tick one box for each question

1. I think the problem of CPE is just a lot of media hype designed to create news stories

	1	2	3	4	5	6	7	8	9	10	
Disagree strongly											Agree strongly

2. I think CPE is a major global health threat that will lead to increasing deaths in future years

	1	2	3	4	5	6	7	8	9	10	
Disagree strongly											Agree strongly

3. I think the consequences of CPE infection for me would be severe

	1	2	3	4	5	6	7	8	9	10	
Disagree strongly											Agree strongly

4. I think the consequences of CPE infection for vulnerable hospital patients would be severe

	1	2	3	4	5	6	7	8	9	10	
Disagree strongly											Agree strongly

5. I think CPE screening is likely to reduce the risk of infection in hospitals

	1	2	3	4	5	6	7	8	9	10	
Disagree strongly											Agree strongly

6. I think screening all hospital patients for CPE is likely to benefit me personally

	1	2	3	4	5	6	7	8	9	10	
Disagree strongly											Agree strongly

7. I think screening all hospital patients for CPE is likely to benefit wider society

	1	2	3	4	5	6	7	8	9	10	
Disagree strongly											Agree strongly

8. As a patient, I think it would be my responsibility to be screened for CPE

	1	2	3	4	5	6	7	8	9	10	
Disagree strongly											Agree strongly

One way of preventing the spread of CPE is by our nurses and doctors checking or screening for CPE when people are admitted to hospital. Patients are asked three questions which allow the nurses to decide if the patient is likely to be carrying CPE. If the answer to any of the questions asked is yes, the nurse will then do a test to confirm if this is the case.

To test for CPE a small sample of faeces or poo is required. The sample is usually taken from a patient's back passage or rectum by a nurse, using a swab which looks similar to a long cotton-bud; a rectal swab. If this is not possible faeces can be collected in a bedpan when a patient goes to the toilet; called a stool sample. This section asks your views about giving a rectal swab or stool sample.

To answer the questions please tick one box for each question

1. Have you ever had a rectal swab or rectal examination by a Doctor or Nurse?

Yes	No

2. Have you ever provided a stool sample?

Yes	No

3. If I needed to be tested for CPE, I would prefer to provide a

Rectal swab?	Stool specimen?	I have no preference

4. If a rectal swab was required to test you for CPE, would you prefer

A nurse took the swab?	You did this yourself?	I have no preference

5. If I was told how to take a rectal swab properly I would prefer to do this myself

Disagree strongly	**1**	**2**	**3**	**4**	**5**	**6**	**7**	**8**	**9**	**10**	Agree strongly

6. If I needed to be tested for CPE I would find having a rectal swab embarrassing

Disagree strongly	**1**	**2**	**3**	**4**	**5**	**6**	**7**	**8**	**9**	**10**	Agree strongly

7. If I needed to be tested for CPE I would feel confident about taking the rectal swab myself

Disagree strongly	**1**	**2**	**3**	**4**	**5**	**6**	**7**	**8**	**9**	**10**	Agree strongly

For most patients carrying CPE it does not cause them any health problems However, it is possible that CPE could spread to other patients within hospitals. For some patients it could cause infections that are very difficult to treat and could be life threatening. To prevent this from happening, patients who are found to be carrying CPE could be cared for in a room by themselves. This section asks your views about the care of a patient who is carrying CPE.

To answer the questions please tick one box for each question

1. I would be worried if I was told I was carrying CPE

Disagree strongly	**1**	**2**	**3**	**4**	**5**	**6**	**7**	**8**	**9**	**10**	Agree strongly

2. If a patient is carrying CPE it's not their fault

Disagree strongly	**1**	**2**	**3**	**4**	**5**	**6**	**7**	**8**	**9**	**10**	Agree strongly

3. If I was carrying CPE I would feel worried about passing on CPE to another person

Disagree strongly	**1**	**2**	**3**	**4**	**5**	**6**	**7**	**8**	**9**	**10**	Agree strongly

4. If I was carrying CPE I would feel lonely if I was placed in a single room

Disagree strongly	**1**	**2**	**3**	**4**	**5**	**6**	**7**	**8**	**9**	**10**	Agree strongly

5. If I was carrying CPE I would prefer the privacy of a single room

Disagree strongly	**1**	**2**	**3**	**4**	**5**	**6**	**7**	**8**	**9**	**10**	Agree strongly

6. I would worry that others might think I am 'dirty' If I was carrying CPE

Disagree strongly	**1**	**2**	**3**	**4**	**5**	**6**	**7**	**8**	**9**	**10**	Agree strongly

7. I would be concerned that I might not get as good healthcare if I was carrying CPE

Disagree strongly	**1**	**2**	**3**	**4**	**5**	**6**	**7**	**8**	**9**	**10**	Agree strongly

This last section asks you about your views on the acceptability of CPE screening.

To answer the questions please tick one box for each question

1. If I were to be admitted to hospital, I would find rectal swabbing for CPE acceptable

Disagree strongly	1	2	3	4	5	6	7	8	9	10	Agree strongly

2. If I were to be admitted to hospital and found to be carrying CPE, I would find being placed in a single room acceptable

Disagree strongly	1	2	3	4	5	6	7	8	9	10	Agree strongly

3. If I were to be admitted to hospital, I would find CPE screening acceptable

Disagree strongly	1	2	3	4	5	6	7	8	9	10	Agree strongly

4. I believe that family members or friends would want me to be screened for CPE if I were to be admitted to hospital

Disagree strongly	1	2	3	4	5	6	7	8	9	10	Agree strongly

5. If a family member or friend was admitted to hospital I would encourage them to be screened for CPE

Disagree strongly	1	2	3	4	5	6	7	8	9	10	Agree strongly

6. I believe that CPE Screening would be acceptable to most people being admitted to hospital

Disagree strongly	1	2	3	4	5	6	7	8	9	10	Agree strongly

7. If I was admitted to hospital careful explanation about CPE screening from a health professional would make screening more acceptable to me

Disagree strongly	1	2	3	4	5	6	7	8	9	10	Agree strongly

Thank you for completing this survey
Please post it back to us in the pre-paid envelope provided.
