# Supplementary material for: The acceptability of screening for Carbapenemase Producing Enterobacteriaceae (CPE): cross-sectional survey of nursing staff and the general publics’ perceptions
Source: Antimicrob Resist Infect Control. 2018 Nov 23;7:144. doi: 10.1186/s13756-018-0434-x (PMC6260859; doi:10.1186/s13756-018-0434-x)
Supplement: Supplementary file 2 — Univariate analysis. (DOCX 23 kb) [file 13756_2018_434_MOESM2_ESM.docx]

## Significant associations between the acceptability of CPE screening and independent variables for Nursing staff

| **Questionnaire Item** | **TDF** |  | **Frequencies (%)**  **I believe that CPE screening is acceptable (Dependant variable)**  **Agree Fail to agree** | **n** | **Chi Square**  **P value** |
| --- | --- | --- | --- | --- | --- |
| I am aware that CPE is an emerging multi-drug resistant bacteria of growing concern | **Knowledge** | **Agree**  **Fail to agree** | 257/300 (85.7) 50/132 (37.9)  43/300 (14.3) 82/132 (62.1) | 432 | χ^2^ =101.8  p<.001 |
| I have been informed about my hospital’s policy and processes for screening patients for CPE | **Knowledge** | **Agree**  **Fail to agree** | 219/296 (74.0) 28/133 (21.1)  77/296 (26.0) 105/133 (78.9) | 429 | χ^2^ =105.3  p<.001 |
| Screening for CPE is undertaken in the clinical area I work in | **Environment** | **Agree**  **Fail to agree** | 260/297 (87.5) 79/131 (60.3)  37/297 (12.5) 52/131 (39.7) | 428 | χ^2^ =40.9  p<.001 |
| The consequences of CPE infection for the patients I care for is/will be so severe that screening will always be a priority | **Beliefs about consequences** | **Agree**  **Fail to agree** | 196/296 (66.2) 14/133 (10.5)  100/296 (33.8) 119/133 (89.5) | 429 | χ^2^ = 113.9  p<.001 |
| Screening patients for CPE would be / is embarrassing for them if a rectal swab is/was required | **Emotion** | **Agree**  **Fail to agree** | 228/302 (75.5) 72/133 (54.1)  74/302 (24.5) 61/133 (45.9) | 435 | χ^2^ =19.7  p<.001 |
| If a rectal swab is/was required as part of CPE screening for the patient I care for they should be asked to do this themselves, if they are able | **Social influences** | **Agree**  **Fail to agree** | 253/301 (84.1) 79/133 (59.4)  48/301 (15.9) 54/133 (40.6) | 434 | χ^2^ =31.2  p<.001 |
| Screening patients for CPE is/would be embarrassing for me as I may need to ask to take a rectal swab | **Emotion** | **Agree**  **Fail to agree** | 67/303 (22.1) 23/133 (17.3)  236/303 (77.9) 110/133 (82.7) | 436 | χ^2^ =1.3  p=0.252 |
| I intend/would intend to conduct CPE screening, on patients on admission, according to my hospital policy | **Intentions** | **Agree**  **Fail to agree** | 291/298 (97.7) 89/133 (66.9)  7/298 (2.3) 44/133 (33.1) | 431 | χ^2^ =83.3  p<.001 |

## Significant associations between the acceptability of CPE screening, demographic and independent variables for the general public

| **If I were to be admitted to hospital I would find CPE screening acceptable** | **Median (IQR)** | **Significance** |
| --- | --- | --- |
| **Worked as a healthcare professional?** | |  |
| Yes (n=43) | 10.0 (2.0) | U= 3368.0  P<.05 |
| No (n=184) | 8.5 (3.0) |  |

| **Have you ever heard about the problem of some bacteria becoming resistant to antibiotics?** | | |
| --- | --- | --- |
| Yes (n=55) | 9.0 (2.0) | U= 2470.0  P<.01 |
| No (n=172) | 8.5 (3.0) |  |
| **Have you heard of CPE?** | | |
| Yes (n=52) | 10.0 (2.0) | U= 3876.0  P<.05 |
| No (n=180) | 8.0 (3.0) |  |
| **Have you ever provided a stool sample** | | |
| Yes (n=111) | 9.0 (2.0) | U= 5402.5  P<.01 |
| No (n=116) | 8.0 (3.75) |  |
| You did this yourself (n=105) | 9.0 (3.0) |  |
| I have no preference (n=85) | 9.0 (3.0) |  |

| **Correlations between independent variables and acceptability for general public** | | |
| --- | --- | --- |
|  | | If I were to be admitted to hospital I would find CPE screening acceptable |
| **Knowledge:**  I think the problem of CPE is just a lot of media hype designed to create news stories | Spearman’s Rho | -.26^**^ |
|  | Sig. (2-tailed) | p<.001 |
|  | N | 225 |
| **Consequences** | Spearman’s Rho | .48^**^ |
|  | Sig. (2-tailed) | p<.001 |
|  | N | 219 |
| **Optimism:**  I think CPE screening is likely to reduce the risk of infections in hospitals | Spearman’s Rho | .44^**^ |
|  | Sig. (2-tailed) | p<.001 |
|  | N | 228 |
| **Social Influence** | Spearman’s Rho | .657^**^ |
|  | Sig. (2-tailed) | p=0.000 |
|  | N | 229 |
| **Capability** | Spearman’s Rho | .160^*^ |
|  | Sig. (2-tailed) | p=0.014 |
|  | N | 237 |
| **Emotions (Screening):**  If I needed to be tested for CPE I would find having a rectal swab embarrassing | Spearman’s Rho | -.179^**^ |
|  | Sig. (2-tailed) | p=0.005 |
|  | N | 239 |
| **Emotions (Isolation)**: If I was carrying CPE I would feel lonely if I was placed in a single room | Spearman’s Rho | -.355^**^ |
|  | Sig. (2-tailed) | p=0.000 |
|  | N | 238 |
| **Emotions (Isolation)**: If I was carrying CPE I would prefer the privacy of a single room | Spearman’s Rho | .365^**^ |
|  | Sig. (2-tailed) | p=0.000 |
|  |  | 236 |
| **Stigma** | Spearman’s Rho | -.219^**^ |
|  | Sig. (2-tailed) | p=.001 |
|  | N | 236 |
| **Acceptability:** If I were to be admitted to hospital, I would find rectal swabbing for CPE acceptable | Spearman’s Rho | .631^**^ |
|  | Sig. (2-tailed) | p=0.000 |
|  | N | 240 |
| **Acceptability:** If I were to be admitted to hospital and found to be carrying CPE, I would find being place in a single room acceptable | Spearman’s Rho | .710^**^ |
|  | Sig. (2-tailed) | p=0.000 |
|  | N | 238 |
| **Acceptability:** If I was admitted to hospital careful explanation about CPE screening from a health professional would make screening more acceptable to me | Spearman’s Rho | .651^**^ |
|  | Sig. (2-tailed) | p=0.000 |
|  | N | 239 |
| **Acceptability:** I believe that CPE screening would be acceptable to most people being admitted to hospital | Spearman’s Rho | .521^**^ |
|  | Sig. (2-tailed) | p=0.000 |
|  | N | 240 |
| **. Correlation is significant at the 0.01 level (2-tailed). *. Correlation is significant at the 0.05 level (2-tailed). | | |
